# Could initial CT chest manifestation in patients hospitalized with COVID 19 pneumonia predict outcome on short term basis

**DOI:** 10.1097/MD.0000000000034115

**Published:** 2023-06-23

**Authors:** Eman F. Dola, Osama Lamie Nakhla, Mona Gamalludin Alkaphoury

**Affiliations:** a Radiology Department, Faculty of Medicine, Ain Shams University; b Radiology Department, Faculty of Medicine, Beni Sueif University.

**Keywords:** COVID-19 pneumonia, CT chest, short interval follow up

## Abstract

Chest computed tomography (CT) can be used to monitor the course of the disease or response to therapy. Therefore, our study was designed to identify chest CT manifestations that can predict the outcome of patients on short term follow-up. This was a retrospective study wherein we reviewed chest CT scans of 112 real-time reverse transcription polymerase chain reaction positive patients admitted to our hospital. All 112 patients underwent follow-up chest CT at a time interval of 4 to 42 days. Our study included 83 male and 29 female who were positive for COVID 19 infection and admitted to the hospital with positive chest CT findings. All patients underwent follow-up chest CT, and the outcomes were categorized as resolution, regression, residual fibrosis, progression, or death. These proportions were 5.4%, 48.2%, 24.1%, 14.3%, and 8%, respectively. The only significant factor in determining the complete resolution of chest CT was oligo-segmental affection (*P* = .0001). The main CT feature that significantly affected the regression of chest CT manifestations was diffuse nodular shadows (*P* = .039). The CT features noted in patients with residual fibrosis were interstitial thickening, with a *P* value of .017. The mono-segmental process significantly affected progression (*P* = .044). The significant factors for fatality were diffuse crazy paving, pleural effusion, and extra-thoracic complications (*P* = .033, .029, and .007, respectively). The prognostic value of the first admission CT can help assess disease outcomes in the earliest phases of onset. This can improve resource distribution.

**Key points**

- COVID 19 infected patient and admitted with positive chest CT findings.
- Short interval follow up.
- CT chest prognostic value.

## 1. Introduction

Since the coronavirus disease 2019 (COVID-19) outbreak started in Wuhan, China, in December 2019 as the first human case infected, the virus has rapidly spread with human-to-human transmission despite imposed precautions and was announced as a pandemic by the WHO Health Organization on March 12, 2020.^[1]^ Chest computed tomography (CT) is an easy, rapid, and noninvasive modality for the diagnosis of pneumonia, with a sensitivity of 98% for the diagnosis of patients with COVID-19 pneumonia.^[2]^ CT chest repetition is allowed in suspected cases with complications; however, in recovering patients, chest CT is not the modality of choice.^[3]^ Chest CT can be used to monitor the course of the disease or the response to therapy.^[4]^ Recent studies have shown that about 94% of COVID 19 pneumonia patients showed lung manifestation at the last scan before discharge,^[5]^ while half of the patients with mild severity COVID 19 pneumonias resolve within 3 weeks.^[6]^

Other modalities are available to assess patients with COVID infection, they have many limitations. For example, chest radiography proved to be of limited value in the diagnosis of early stages, especially in mild disease courses, compared to the intermediate to advanced stages of COVID-19 with features of acute respiratory distress syndrome (ARDS) and follow-up.^[7]^ Benmalek et al^[8]^ reported a higher sensitivity of chest CT than chest radiography in detecting positive cases of COVID pneumonia.

Another modality that could be considered is Chest US; however, it also has limitations in the diagnosis of COVID pneumonia. According to Colombi et al,^[9]^ admission chest CT shows better performance than Lung US for COVID-19 diagnosis at varying disease prevalence. Chest US showed high sensitivity but was not specific for COVID-19.

Patients survived COVID 19 infections either recover completely, develop functional impairment in the form of persistent symptoms, or develop abnormalities after full recovery.^[10]^

In our study, the patient underwent CT at admission with follow-up CT before discharge or to follow-up complications with a time interval of 4 to 42 days (mean 11.60 days ± standard deviation [SD] 7.95). All chest CT manifestations at the time of admission were recorded, and in follow-up CT, we stratified patients with complete resolution, regression, Residual fibrotic pattern, progression, or death.

The aim of our study was to detect chest CT manifestations that can predict the outcome of patients during short-term follow-up. The prognostic value of the first admission CT can help assess disease outcomes in the earliest phases of onset. This can improve resource distribution.

## 2. Materials and methods

This was a retrospective study wherein we reviewed the chest CT scans of 112 real-time reverse transcription polymerase chain reaction positive patients at our hospital from April 2021 to December 2021. All patients underwent chest CT at admission, followed by chest CT just before discharge or with the development of complications. The study was approved by the Institutional Ethical committee. The requirement for informed patient consent was waived.

### 2.1. Study design and participants

This retrospective study was approved by the ethics committee of the Ain Shams University. All participants were anonymous. Initial chest CT was performed on the first day of admission. All 112 patients underwent follow-up chest CT with a time interval of 4 to 42 days (mean 11.60 days ± SD 7.95) using the same CT chest scanner and the same protocol. All patients underwent follow-up CT before discharge due to the short hospital stay and the need to confirm complete resolution before being released from the hospital. The remaining patients who had regression or progression, chest CT was performed as part of the follow-up.

All CT images were reviewed by 3 senior cardiothoracic radiologists (with 14, 7, and 6 years of experience in thoracic radiology). The readers independently assessed the CT features using the axial and multiplanar reconstructed images. The predominant CT patterns were as follows: ground-glass opacities, consolidation, halo sign, crazy paving, reticulation, subpleural line, fibrotic strips, presence of nodules, bronchiectasis, pleural effusion, pleural thickening, and Lymph Nodes. Then, lobar affection as multi-segmental, oligo-segmental, or mono-segmental was stratified followed by zonal affection either upper, lower, or diffuse distribution.

CT evidence of residual fibrotic-like changes was defined as the presence of residual reticulation, ground glass, and fibrotic-like changes.

**Figure 1.**
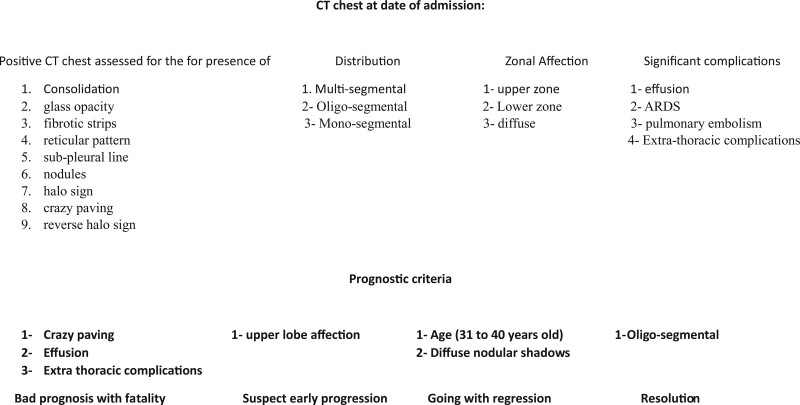
Assessment flow chart. CT = computed tomography.

Chest CT manifestations recorded on the first day were compared with the follow-up CT at discharge. The findings affecting the outcome were tabulated, and their significance was calculated. The assessment system was built for future CT chest evaluations at the time of admission for COVID 19 infected patient (Fig. 1).

### 2.2. Imaging protocol

All participants with a positive PCR test for COVID-19 underwent chest CT using a multi-slice 128 CT machine (Optima CT 660; GE, Boston, MA). Axial high-resolution lung tissue cuts were made with a slice thickness of 0.63 cm with and a GAP of 4.38. The axial lung and mediastinal windows were imaged with a slice thickness of 5 mm and a GAP of 0. Coronal and sagittal reconstructions were performed in the lung and mediastinal windows.

### 2.3. Data management and statistical analysis

The collected data were revised, coded, tabulated, and introduced to a PC using the Statistical Package for Social Sciences (IBM SPSS 20.0). Data are presented, and suitable analysis was performed according to the type of data obtained for each parameter.

### 2.4. Descriptive statistics

Mean ± SD and range for parametric numerical data, and median and interquartile range for non-parametric data.

#### 2.4.1. Analytical statistics:

**Chi square test** was used to examine the relationship between 2 qualitative variables; however, when the expected count was less than 5 in more than 20% of the cells, Fisher’s exact test was used.

***P* value: level of significance:** *P* > .05: non-significant; *P* < .05: significant; *P* < .01: highly significant.

## 3. Results

Our study included 83 male and 29 female who were positive for COVID 19 infection and admitted to the hospital with positive chest CT findings. Their ages ranged from 31 to 60 years as shown (Table 1).

**Table 1:** Characteristics of the studied patients (n = 112).

Variables		No.	%
Age	Less than or equal 30 yr	11	9.9
	31–40 yr	34	30.6
	41–50 yr	22	19.8
	51–60 yr	31	27.9
	More than 60 yr	13	11.7
Sex	Male	83	74.1
	Female	29	25.9

The positive radiological findings were ground glass opacity in 86.4% of our patients, followed by consolidations in 44.6%, fibrotic strips in 51.8%, reticular pattern in 26.8%, subpleural line in 22.3%, and nodules in 17.9%. The least common findings were the halo sign, which was seen in 17 patients, crazy paving in 6 patients, and reverse halo sign in only5 patients.

Regarding distribution, 81.2% of our study population showed multi-segmental affection, with nearly half of the patients showing diffuse zonal affection, and more than one-third of the patients showed lower zone predominance (Table 2).

**Table 2:** Radiological findings among PCR +ve patients (n = 112).

Variables		No.	%
Reverse Halo	Negative	107	95.5
	Positive	5	4.5
Ground Glass Opacity	Negative	15	13.5
	Diffuse	49	44.1
	Central	4	3.6
	Subpleural	43	38.7
Consolidation	Negative	62	55.4
	Diffuse	22	19.6
	Central	2	1.8
	Subpleural	26	23.2
Halo sign	Negative	95	84.8
	Positive	17	15.2
Crazy Paving	Negative	106	94.6
	Diffuse	5	4.5
	Lobar	1	0.9
	Subpleural	0	0.0
Interstitial Thickening	Negative	82	73.2
	Positive	30	26.8
Subpleural lines	Negative	87	77.7
	Positive	25	22.3
Fibrotic Strips	Negative	54	48.2
	Positive	58	51.8
Nodules	Negative	92	82.1
	Positive	20	17.9
Site	Negative	92	82.1
	Peri-bronchial nodules	7	6.2
	Diffuse nodules	3	2.7
	Subpleural nodules	10	8.9
Vascular Enlargement	Negative	111	99.1
	Positive	1	0.9
Bronchial Dilatation	Negative	111	99.1
	Positive	1	0.9
Lobar Affection	Negative	0	0.0
	Multilobar	91	81.2
	Oligosegmental	17	15.2
	Monosegmental	4	3.6
Zonal Affection	Negative	0	0.0
	Diffuse	62	55.4
	Lower	42	37.5
	Upper	8	7.1
Other Pulmonary findings	Negative	109	97.3
	Cavitation + Pulmonary embolism	1	0.9
	Pneumothorax and Pneumomediastinum	1	0.9
	Bilateral Subpleural Calcific nodules	1	0.9
Pleural findings	Negative	104	92.9
	Pleural thickening	3	2.7
	Pleural Effusion	5	4.5
Lymph nodes	Negative	111	99.1
	Positive	1	0.9

PCR = polymerase chain reaction.

Only 2 patients had other findings not related to COVID 19 infection that including pneumothorax and pneumomediastinum (post-intubation) and sub-pleural calcific nodules.

Complications of COVID 19 were seen as effusion in 5 patients (secondary to on-top bacterial infections) and 1 patient developed pulmonary embolism. Extra thoracic complications included pericardial effusion, hemorrhagic encephalitis, and multi-organ failure (Table 3).

**Table 3:** Fate among PCR +ve patients (n = 112).

Variables		No.	%
Fate	Resolution	6	5.4
	Regression	54	48.2
	Residual Fibrotic	27	24.1
	Progression	16	14.3
	Death	9	8.0

PCR = polymerase chain reaction.

All patients underwent follow-up CT either before discharge to confirm the resolution of the disease, as part of follow-up, or with development of complications.

The outcomes were categorized as resolution, regression, residual fibrosis, progression, or death. These proportions were 5.4%, 48.2%, 24.1%, 14.3%, and 8%, respectively (Table 4).

**Table 4:** Extra-thoracic complications among PCR +ve patients (n = 112).

Variables		No.	%
Extrathorathic complications	Negative	109	97.3
	Pericardial effusion	1	0.9
	Liver failure, renal failure, Ascites with edema	1	0.9
	bifrontal Hemorrhagic encephalitis	1	0.9

PCR = polymerase chain reaction.

We found that 40% of patients admitted with diffuse crazy paving ended by death, which represents a significant factor with a *P* value of .033. Pleural effusion developed in about 40% of the patients who died and was a significant factor in fatality (*P* = .029). All patients who developed extrathoracic complications apart from pericardial effusion ended with death, all of which were significant factors (*P* = .007) (Table 5).

**Table 5:** Factors determining Poor fate (death) in the studied patients (n = 112).

Variables		Death				Chi-square test	*P* value
		Negative		Positive			
		No.	%	No.	%		
Age	Less than or equal 30 yr	11	100.0	0	0.0	5.601 FE (#)	.135
	31–40 yr	34	100.0	0	0.0		
	41–50 yr	21	95.5	1	4.5		
	51–60 yr	28	90.3	3	9.7		
	More than 60 yr	11	84.6	2	15.4		
Sex	Male	78	94.0	5	6.0	0.281 FE (#)	1.000
	Female	28	96.6	1	3.4		
Reverse Halo	Negative	101	94.4	6	5.6	0.296 FE (#)	1.000
	Positive	5	100.0	0	0.0		
Ground Glass Opacity	Negative	15	100.0	0	0.0	1.311 FE (#)	.693
	Diffuse	45	91.8	4	8.2		
	Central	4	100.0	0	0.0		
	Subpleural	41	95.3	2	4.7		
Consolidation	Negative	59	95.2	3	4.8	1.690 FE (#)	.754
	Diffuse	20	90.9	2	9.1		
	Central	2	100.0	0	0.0		
	Subpleural	25	96.2	1	3.8		
Halo sign	Negative	89	93.7	6	6.3	1.134 FE (#)	.588
	Positive	17	100.0	0	0.0		
Crazy Paving	Negative	102	96.2	4	3.8	8.482 FE (#)	.033**
	Diffuse	3	60.0	2	40.0		
	Lobar	1	100.0	0	0.0		
	Subpleural	0	0.0	0	0.0		
Interstitial Thickening	Negative	78	95.1	4	4.9	0.139 FE (#)	.658
	Positive	28	93.3	2	6.7		
Subpleural lines	Negative	83	95.4	4	4.6	0.443 FE (#)	.614
	Positive	23	92.0	2	8.0		
Fibrotic Strips	Negative	51	94.4	3	5.6	0.008 FE (#)	1.000
	Positive	55	94.8	3	5.2		
Nodules	Negative	86	93.5	6	6.5	1.378 FE (#)	.589
	Positive	20	100.0	0	0.0		
Site	Negative	86	93.5	6	6.5	0.702	1.000
	Peri-bronchial nodules	7	100.0	0	0.0		
	Diffuse nodules	3	100.0	0	0.0		
	Subpleural nodules	10	100.0	0	0.0		
Vascular Enlargement	Negative	105	94.6	6	5.4	0.057 FE (#)	1.000
	Positive	1	100.0	0	0.0		
Bronchial Dilatation	Negative	105	94.6	6	5.4	0.057 FE (#)	1.000
	Positive	1	100.0	0	0.0		
Lobar Affection	Negative	0	0.0	0	0.0	0.382 FE (#)	.670
	Multilobar	85	93.4	6	6.6		
	Oligosegmental	17	100.0	0	0.0		
	Monosegmental	4	100.0	0	0.0		
Zonal Affection	Negative	0	0.0	0	0.0	2.308 FE (#)	.228
	Diffuse	60	96.8	2	3.2		
	Lower	39	92.9	3	7.1		
	Upper	7	87.5	1	12.5		
Other Pulmonary findings	Negative	104	95.4	5	4.6	9.498 FE (#)	.154
	Cavitation + Pulmonary embolism	0	0.0	1	100.0		
	Pneumothorax and Pneumomediastinum	1	100.0	0	0.0		
	Bilateral Subpleural Calcific nodules	1	100.0	0	0.0		
Pleural findings	Negative	100	96.2	4	3.8	7.558 FE (#)	.029*
	Pleural thickening	3	100.0	0	0.0		
	Pleural Effusion	3	60.0	2	40.0		
Lymph nodes	Negative	106	95.5	5	4.5	17.826 FE (#)	.054
	Positive	0	0.0	1	100.0		
Extrathorathic Complications	Negative	105	96.3	4	3.7	15.587 FE (#)	.007**
	Pericardial effusion	1	100.0	0	0.0		
	Liver failure, renal failure, Ascites with edema	0	0.0	1	100.0		
	bifrontal Hemorrhagic encephalitis	0	0.0	1	100.0		

* (significant).

** (Highly significant).

The total fatality rate in our study was 9 patients, all of whom showed diffuse ground glass opacities, 6 of whom showed diffuse interstitial thickening, mainly sub-pleural with fibrotic strips and sub-pleural lines, 4 patients showed crazy paving at the time of admission, 2 patients showed consolidations mainly peripheral, 3 patients developed effusion, one had cavitation and pulmonary embolism. The cause of death was mainly the development of ARDS, which was seen in 6 out of 9 patients. The other 3 died of extra-thoracic complications, including hemorrhagic encephalitis and multi-organ failure.

The only significant factor in determining the complete resolution of chest CT was oligo-segmental affection (*P* = .0001) (Table 6).

**Table 6:** Factors affecting resolution in the studied patients (n = 112).

Variables		Resolution				Chi square test	*P* value
		Negative		Positive			
		No.	%	No.	%		
Age	Less than or equal 30 yr	9	81.8	2	18.2	4.360 FE (#)	.275
	31–40 yr	32	94.1	2	5.9		
	41–50 yr	22	100.0	0	0.0		
	51–60 yr	29	93.5	2	6.5		
	More than 60 yr	13	100.0	0	0.0		
Sex	Male	79	95.2	4	4.8	0.183 FE (#)	.648
	Female	27	93.1	2	6.9		
Reverse Halo	Negative	101	94.4	6	5.6	0.296 FE (#)	1.000
	Positive	5	100.0	0	0.0		
Ground Glass Opacity	Negative	14	93.3	1	6.7	0.779 FE (#)	1.000
	Diffuse	46	93.9	3	6.1		
	Central	4	100.0	0	0.0		
	Subpleural	41	95.3	2	4.7		
Consolidation	Negative	56	90.3	6	9.7	4.304 FE (#)	.222
	Diffuse	22	100.0	0	0.0		
	Central	2	100.0	0	0.0		
	Subpleural	26	100.0	0	0.0		
Halo sign	Negative	89	93.7	6	6.3	1.134 FE (#)	.588
	Positive	17	100.0	0	0.0		
Crazy Paving	Negative	100	94.3	6	5.7	1.410 FE (#)	1.000
	Diffuse	5	100.0	0	0.0		
	Lobar	1	100.0	0	0.0		
	Subpleural	0	0.0	0	0.0		
Interstitial Thickening	Negative	76	92.7	6	7.3	2.319 FE (#)	.190
	Positive	30	100.0	0	0.0		
Subpleural lines	Negative	82	94.3	5	5.7	0.117 FE (#)	1.000
	Positive	24	96.0	1	4.0		
Fibrotic Strips	Negative	49	90.7	5	9.3	3.132 FE (#)	.104
	Positive	57	98.3	1	1.7		
Nodules	Negative	87	94.6	5	5.4	0.006 FE (#)	1.000
	Positive	19	95.0	1	5.0		
Site	Negative	87	94.6	5	5.4	1.445 FE (#)	.702
	Peri-bronchial nodules	7	100.0	0	0.0		
	Diffuse nodules	3	100.0	0	0.0		
	Subpleural nodules	9	90.0	1	10.0		
Vascular Enlargement	Negative	105	94.6	6	5.4	0.057 FE (#)	1.000
	Positive	1	100.0	0	0.0		
Bronchial Dilatation	Negative	105	94.6	6	5.4	0.057 FE (#)	1.000
	Positive	1	100.0	0	0.0		
Lobar Affection	Negative	0	0.0	0	0.0	22.620 FE (#)	.000**
	Multilobar	91	100.0	0	0.0		
	Oligosegmental	11	64.7	6	35.3		
	Monosegmental	4	100.0	0	0.0		
Zonal Affection	Negative	0	0.0	0	0.0	4.691 FE (#)	.107
	Diffuse	61	98.4	1	1.6		
	Lower	37	88.1	5	11.9		
	Upper	8	100.0	0	0.0		
Other Pulmonary findings	Negative	103	94.5	6	5.5	3.792 FE (#)	1.000
	Cavitation + Pulmonary embolism	1	100.0	0	0.0		
	Pneumothorax and Pneumomediastinum	1	100.0	0	0.0		
	Bilateral Subpleural Calcific nodules	1	100.0	0	0.0		
Pleural findings	Negative	98	94.2	6	5.8	0.565 FE (#)	1.000
	Pleural thickening	3	100.0	0	0.0		
	Pleural Effusion	5	100.0	0	0.0		
Lymph nodes	Negative	105	94.6	6	5.4	0.057 FE (#)	1.000
	Positive	1	100.0	0	0.0		
Extrathorathic Complications	Negative	103	94.5	6	5.5	3.792 FE (#)	1.000
	Pericardial effusion	1	100.0	0	0.0		
	Liver failure, renal failure, Ascites with edema	1	100.0	0	0.0		
	bifrontal Hemorrhagic encephalitis	1	100.0	0	0.0		

* (significant).

** (Highly significant).

Six of the 112 patients who showed complete resolution of CT after short-term follow-up had sub-pleural ground-glass opacity, one showed sub-pleural ground-glass nodules, and one showed Sub-pleural lines associated with sub-pleural ground-glass opacities.

Patients with regressive chest CT manifestations were more common in the 31 to 40 years group, with a significant *P* value of .022. The main CT feature that significantly affected the regression of chest CT manifestations was diffuse nodular shadows (*P* = .039) (Table 7).

**Table 7:** Factors affecting regression in the studied patients (n = 112).

Variables		Regression				Chi square test	*P* value
		Negative		Positive			
		No.	%	No.	%		
Age	Less than or equal 30 yr	6	54.5	5	45.5	11.459	.022*
	31–40 yr	11	32.4	23	67.6		
	41–50 yr	11	50.0	11	50.0		
	51–60 yr	23	74.2	8	25.8		
	More than 60 yr	7	53.8	6	46.2		
Sex	Male	44	53.0	39	47.0	0.193	.660
	Female	14	48.3	15	51.7		
Reverse Halo	Negative	56	52.3	51	47.7	0.291 FE (#)	.671
	Positive	2	40.0	3	60.0		
Ground Glass Opacity	Negative	10	66.7	5	33.3	2.879 FE (#)	.415
	Diffuse	26	53.1	23	46.9		
	Central	1	25.0	3	75.0		
	Subpleural	20	46.5	23	53.5		
Consolidation	Negative	32	51.6	30	48.4	0.361 FE (#)	1.000
	Diffuse	11	50.0	11	50.0		
	Central	1	50.0	1	50.0		
	Subpleural	14	53.8	12	46.2		
Halo sign	Negative	52	54.7	43	45.3	2.183	.189
	Positive	6	35.3	11	64.7		
Crazy Paving	Negative	53	50.0	53	50.0	2.447 FE (#)	.365
	Diffuse	4	80.0	1	20.0		
	Lobar	1	100.0	0	0.0		
	Subpleural	0	0.0	0	0.0		
Interstitial Thickening	Negative	41	50.0	41	50.0	0.391	.670
	Positive	17	56.7	13	43.3		
Subpleural lines	Negative	45	51.7	42	48.3	0.001	1.000
	Positive	13	52.0	12	48.0		
Fibrotic Strips	Negative	29	53.7	25	46.3	0.154	.710
	Positive	29	50.0	29	50.0		
Nodules	Negative	52	56.5	40	43.5	4.628	.047*
	Positive	6	30.0	14	70.0		
Site	Negative	52	56.5	40	43.5	7.489 FE (#)	.039*
	Peri-bronchial nodules	1	14.3	6	85.7		
	Diffuse nodules	0	0.0	3	100.0		
	Subpleural nodules	5	50.0	5	50.0		
Vascular Enlargement	Negative	58	52.3	53	47.7	1.084 FE (#)	.482
	Positive	0	0.0	1	100.0		
Bronchial Dilatation	Negative	57	51.4	54	48.6	0.939	1.000
	Positive	1	100.0	0	0.0		
Lobar Affection	Negative	0	0.0	0	0.0	1.432	.448
	Multilobar	45	49.5	46	50.5		
	Oligosegmental	11	64.7	6	35.3		
	Monosegmental	2	50.0	2	50.0		
Zonal Affection	Negative	0	0.0	0	0.0	1.225	.578
	Diffuse	31	50.0	31	50.0		
	Lower	24	57.1	18	42.9		
	Upper	3	37.5	5	62.5		
Other Pulmonary findings	Negative	56	51.4	53	48.6	2.748 FE (#)	.865
	Cavitation + Pulmonary embolism	1	100.0	0	0.0		
	Pneumothorax and Pneumomediastinum	1	100.0	0	0.0		
	Bilateral Subpleural Calcific nodules	0	0.0	1	100.0		
Pleural findings	Negative	54	51.9	50	48.1	0.690 FE (#)	.872
	Pleural thickening	1	33.3	2	66.7		
	Pleural Effusion	3	60.0	2	40.0		
Lymph nodes	Negative	57	51.4	54	48.6	0.939 FE (#)	1.000
	Positive	1	100.0	0	0.0		
Extrathorathic Complications	Negative	56	51.4	53	48.6	2.748 FE (#)	.865
	Pericardial effusion	0	0.0	1	100.0		
	Liver failure, renal failure, Ascites with edema	1	100.0	0	0.0		
	bifrontal Hemorrhagic encephalitis	1	100.0	0	0.0		

* (significant).

** (Highly significant).

Of the 54 patients with a regressive course, 49 showed ground glassing, 24showed consolidation, 29 had fibrotic strips, 13 had interstitial thickening, and 12 showed subpleural lines.

Forty-six patients showed multi-segmental affection, 6 oligo-segmental affection, and 2 had mono-segmental affection on chest CT. The other chest CT finding noted was mild effusion in 2 patients.

Only 1 patient had an extra thoracic complication, pericardial effusion, which was resolved on follow-up CT.

The CT features noted in patients with residual fibrosis were interstitial thickening, which was observed in 40% of patients (*P* = .017) (Table 8).

**Table 8:** Factors affecting residual fibrotic in the studied patients (n = 112).

Variables		Residual fibrotic				Chi square test	*P* value
		Negative		Positive			
		No.	%	No.	%		
Age	Less than or equal 30 yr	9	81.8	2	18.2	5.818 FE (#)	.205
	31–40 yr	30	88.2	4	11.8		
	41–50 yr	16	72.7	6	27.3		
	51–60 yr	20	64.5	11	35.5		
	More than 60 yr	9	69.2	4	30.8		
Sex	Male	61	73.5	22	26.5	1.008	.450
	Female	24	82.8	5	17.2		
Reverse Halo	Negative	82	76.6	25	23.4	0.723 FE (#)	.592
	Positive	3	60.0	2	40.0		
Ground Glass Opacity	Negative	8	53.3	7	46.7	5.456 FE (#)	.119
	Diffuse	40	81.6	9	18.4		
	Central	4	100.0	0	0.0		
	Subpleural	32	74.4	11	25.6		
Consolidation	Negative	48	77.4	14	22.6	1.462 FE (#)	.719
	Diffuse	16	72.7	6	27.3		
	Central	1	50.0	1	50.0		
	Subpleural	20	76.9	6	23.1		
Halo sign	Negative	71	74.7	24	25.3	0.457 FE (#)	.759
	Positive	14	82.4	3	17.6		
Crazy Paving	Negative	80	75.5	26	24.5	0.519 FE (#)	1.000
	Diffuse	4	80.0	1	20.0		
	Lobar	1	100.0	0	0.0		
	Subpleural	0	0.0	0	0.0		
Interstitial Thickening	Negative	67	81.7	15	18.3	5.657	.017*
	Positive	18	60.0	12	40.0		
Subpleural lines	Negative	68	78.2	19	21.8	1.096	.300
	Positive	17	68.0	8	32.0		
Fibrotic Strips	Negative	45	83.3	9	16.7	3.155	0.083
	Positive	40	69.0	18	31.0		
Nodules	Negative	68	73.9	24	26.1	1.104 FE (#)	.393
	Positive	17	85.0	3	15.0		
Site	Negative	68	73.9	24	26.1	2.822 FE (#)	.371
	Peri-bronchial nodules	7	100.0	0	0.0		
	Diffuse nodules	3	100.0	0	0.0		
	Subpleural nodules	7	70.0	3	30.0		
Vascular Enlargement	Negative	84	75.7	27	24.3	0.321 FE (#)	1.000
	Positive	1	100.0	0	0.0		
Bronchial Dilatation	Negative	85	76.6	26	23.4	3.177 FE (#)	.241
	Positive	0	0.0	1	100.0		
Lobar Affection	Negative	0	0.0	0	0.0	4.778 FE (#)	.099
	Multilobar	65	71.4	26	28.6		
	Oligosegmental	16	94.1	1	5.9		
	Monosegmental	4	100.0	0	0.0		
Zonal Affection	Negative	0	0.0	0	0.0	3.211 FE (#)	.198
	Diffuse	44	71.0	18	29.0		
	Lower	33	78.6	9	21.4		
	Upper	8	100.0	0	0.0		
Other Pulmonary findings	Negative	83	76.1	26	23.9	3.527 FE (#)	.567
	Cavitation + Pulmonary embolism	1	100.0	0	0.0		
	Pneumothorax and Pneumomediastinum	0	0.0	1	100.0		
	Bilateral Subpleural Calcific nodules	1	100.0	0	0.0		
Pleural findings	Negative	77	74.0	27	26.0	1.668 FE (#)	.323
	Pleural thickening	3	100.0	0	0.0		
	Pleural Effusion	5	100.0	0	0.0		
Lymph nodes	Negative	84	75.7	27	24.3	0.321 FE (#)	1.000
	Positive	1	100.0	0	0.0		
Extrathorathic Complications	Negative	82	75.2	27	24.8	1.281 FE (#)	1.000
	Pericardial effusion	1	100.0	0	0.0		
	Liver failure, renal failure, Ascites with edema	1	100.0	0	0.0		
	bifrontal Hemorrhagic encephalitis	1	100.0	0	0.0		

* (significant).

** (Highly significant).

A total of 27 patients showed residual fibrosis, 20 out of the 27 showed ground glass and 13 out 0f 27 showed consolidations with different distributions. Eighteen patients had fibrotic strips, eight had a subpleural line, three had nodules, and only 1 patient had a crazy paving pattern at the time of admission. Nearly all patients showed multi-segmental affection, except for 1 patient who had oligo-segmental affection. Only 1 patient developed pneumothorax and pneumomediastinum as post-intubation complications.

All patients who showed early Chest CT progressive features after a short-term follow-up finally resolved clinically and were discharged with clinical improvement; however, these patients were not followed up in our study. We found that the mono-segmental process significantly affected progression (*P* = .044) (Table 9).

**Table 9:** Factors affecting progression in the studied patients (n = 112).

Variables		Progression				Chi square test	*P* value
		Negative		Positive			
		No.	%	No.	%		
Age	Less than or equal 30 yr	9	81.8	2	18.2	1.079 FE (#)	.929
	31–40 yr	29	85.3	5	14.7		
	41–50 yr	18	81.8	4	18.2		
	51–60 yr	27	87.1	4	12.9		
	More than 60 yr	12	92.3	1	7.7		
Sex	Male	73	88.0	10	12.0	1.311	.354
	Female	23	79.3	6	20.7		
Reverse Halo	Negative	91	85.0	16	15.0	0.872 FE (#)	1.000
	Positive	5	100.0	0	0.0		
Ground Glass Opacity	Negative	13	86.7	2	13.3	2.015 FE (#)	.499
	Diffuse	41	83.7	8	16.3		
	Central	3	75.0	1	25.0		
	Subpleural	39	90.7	4	9.3		
Consolidation	Negative	54	87.1	8	12.9	4.876 FE (#)	.151
	Diffuse	21	95.5	1	4.5		
	Central	2	100.0	0	0.0		
	Subpleural	19	73.1	7	26.9		
Halo sign	Negative	81	85.3	14	14.7	0.104 FE (#)	1.000
	Positive	15	88.2	2	11.8		
Crazy Paving	Negative	91	85.8	15	14.2	4.343 FE (#)	.161
	Diffuse	5	100.0	0	0.0		
	Lobar	0	0.0	1	100.0		
	Subpleural	0	0.0	0	0.0		
Interstitial Thickening	Negative	69	84.1	13	15.9	0.615	.552
	Positive	27	90.0	3	10.0		
Subpleural lines	Negative	73	83.9	14	16.1	1.038	.517
	Positive	23	92.0	2	8.0		
Fibrotic Strips	Negative	44	81.5	10	18.5	1.526	.217
	Positive	52	89.7	6	10.3		
Nodules	Negative	78	84.8	14	15.2	0.365 FE (#)	.733
	Positive	18	90.0	2	10.0		
Site	Negative	78	84.8	14	15.2	0.393 FE (#)	1.000
	Peri-bronchial nodules	6	85.7	1	14.3		
	Diffuse nodules	3	100.0	0	0.0		
	Subpleural nodules	9	90.0	1	10.0		
Vascular Enlargement	Negative	95	85.6	16	14.4	0.168 FE (#)	1.000
	Positive	1	100.0	0	0.0		
Bronchial Dilatation	Negative	95	85.6	16	14.4	0.168 FE (#)	1.000
	Positive	1	100.0	0	0.0		
Lobar Affection	Negative	0	0.0	0	0.0	5.795 FE (#)	.044*
	Multilobar	81	89.0	10	11.0		
	Oligosegmental	13	76.5	4	23.5		
	Monosegmental	2	50.0	2	50.0		
Zonal Affection	Negative	0	0.0	0	0.0	1.399	.605
	Diffuse	55	88.7	7	11.3		
	Lower	35	83.3	7	16.7		
	Upper	6	75.0	2	25.0		
Other Pulmonary findings	Negative	93	85.3	16	14.7	1.748 FE (#)	1.000
	Cavitation + Pulmonary embolism	1	100.0	0	0.0		
	Pneumothorax and Pneumomediastinum	1	100.0	0	0.0		
	Bilateral Subpleural Calcific nodules	1	100.0	0	0.0		
Pleural findings	Negative	88	84.6	16	15.4	0.444 FE (#)	1.000
	Pleural thickening	3	100.0	0	0.0		
	Pleural Effusion	5	100.0	0	0.0		
Lymph nodes	Negative	95	85.6	16	14.4	0.168 FE (#)	1.000
	Positive	1	100.0	0	0.0		
Extrathorathic Complications	Negative	93	85.3	16	14.7	1.748 FE (#)	1.000
	Pericardial effusion	1	100.0	0	0.0		
	Liver failure, renal failure, Ascites with edema	1	100.0	0	0.0		
	bifrontal Hemorrhagic encephalitis	1	100.0	0	0.0		

* (significant).

** (Highly significant).

Sixteen patients showed progressive chest CT features, 14 out of 16 showed ground glassing opacities, 8 patients showed consolidations, 6/16 showed fibrotic strips, 3 had interstitial marking, 2 showed sub-pleural line, 2 showed halo sign, 2 had nodules and only 1 patient showed central crazy-paving changes. The main distribution was multisegmental, observed in 10 patients. The zonal distribution was equal between diffuse and lower zone affection. None of these patients had pleural effusion, lymph node involvement, or extra-thoracic complications.

## 4. Discussion

In our study, we tried to assess the significance and prognostic value of each chest CT characteristic at the short-term follow-up of patients diagnosed with COVID 19 infections and showed positive results in the initial chest CT at the time of admission.

Laino et al^[11]^ found that the CT scan sensitivity as a prognostic factor was high during the first 2 to 3 weeks after symptom onset. However, Li et al demonstrated that CT prognosis was more significant if it was performed within 6 days of symptom onset. They found that the main prognostic characteristic was in the period of 6 to 10 days. In our study, the time interval was 4 to 42 days.

Liu et al,^[12]^ who followed up patients admitted to their hospital and diagnosed with COVID 19 infection, found that complete resolution was significantly higher in patients younger than 44 years. In our study, no association was found between the age of the patients and complete resolution; however, it was seen as a significant factor in patients with regressive CT chest features (the patient age was between 31 and 40 years old).

**Figure 2.**
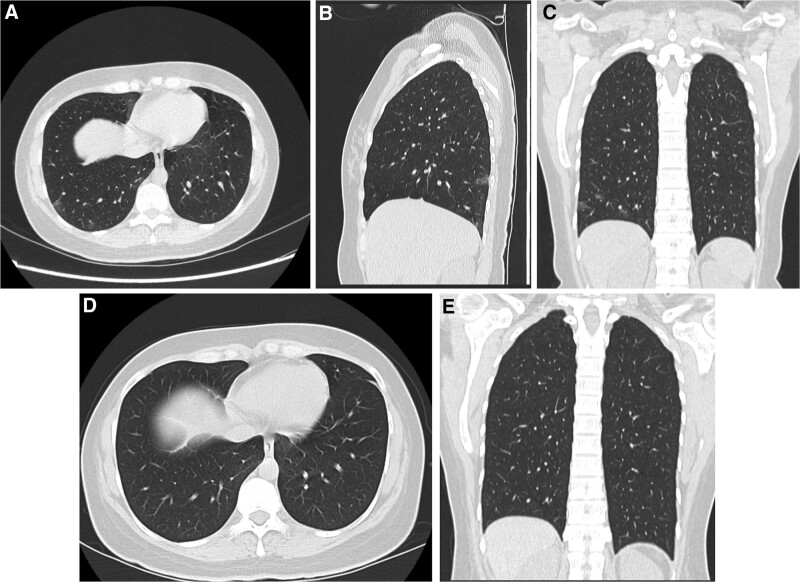
Patients confirmed positive for COVID 19, first day at admission CT chest showed. (A) Axial CT chest cuts showed few ground glassing nodules at basal segments of right lower lung lobe. (B and C) Sagittal and coronal reformate confirmed the ground glassing nodules with oligo-segmental affection. (D and E) Axial and coronal images in follow up after 6 d confirmed complete resolution of CT chest manifestations. CT = computed tomography.

**Figure 3.**
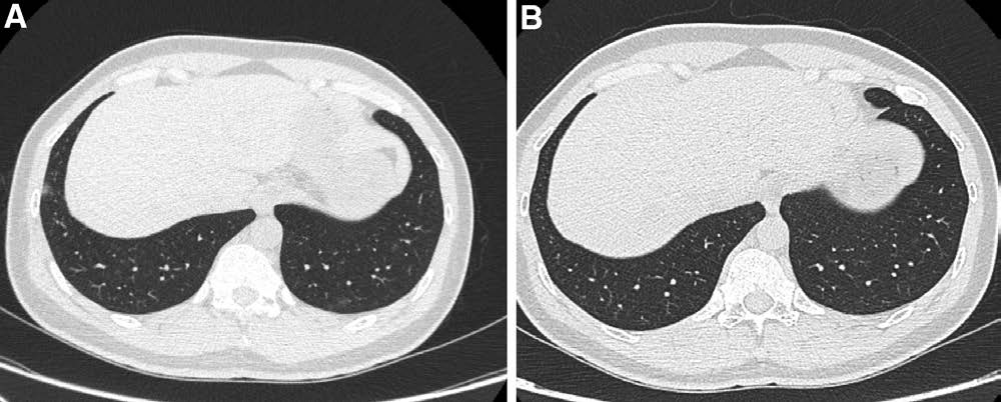
Patients confirmed positive for COVID 19, first day at admission CT chest showed. (A) Axial CT chest cuts showed few ground glassing nodules at basal segments of both lower lung lobe. (B) Axial CT chest cuts in follow up after 8 d confirmed complete resolution of CT chest manifestations. CT = computed tomography.

Another study by Meiler et al^[13]^ found that only 1 chest CT feature associated with favorable outcome was unilateral distribution, which was comparable to our study, which proved oligo-segmental affection as the main significant factor for resolution (Figs. 2 and 3).

**Figure 4.**
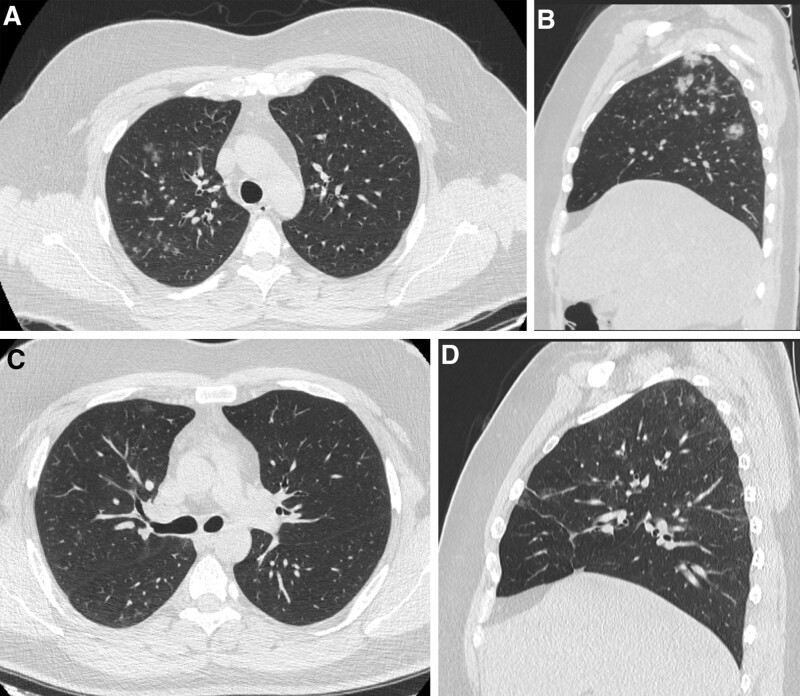
Patients confirmed positive for COVID 19, first day at admission CT chest showed. (A) Axial CT chest cuts showed multiple right upper lung lobe nodules. (B) Sagittal reformate confirmed the right upper and lower lung lobes. (C and D) Axial and sagittal images in follow up after 18 d confirmed regression of the CT chest manifestations with residual faint ground glassing nodules. CT = computed tomography.

We also proved that diffuse nodular shadows were another significant factor in determining regression, with a *P* value of .047. This could be attributed to the extent of parenchymal involvement, as nodular shadows could represent an early manifestation of COVID-19 pneumonia, so early less parenchymal involvement could lead to regression of the disease process (Fig. 4).

The main significant feature that showed a significant *P* value in patients who died was diffuse crazy paving followed by pleural effusion, considering both as poor prognostic factors. This agrees with the findings of Erturk et al,^[14]^ who found that the significant prognostic factors associated with ICU admission were crazy paving and enlargement of the mediastinal hilar node. Also, Parry eta al,^[15]^ divided the patient into either stable or non-stable. They found that bilateral central involvement was more significant in unstable patients, and 70% of clinically unstable patients showed a crazy paving pattern.

**Figure 5.**
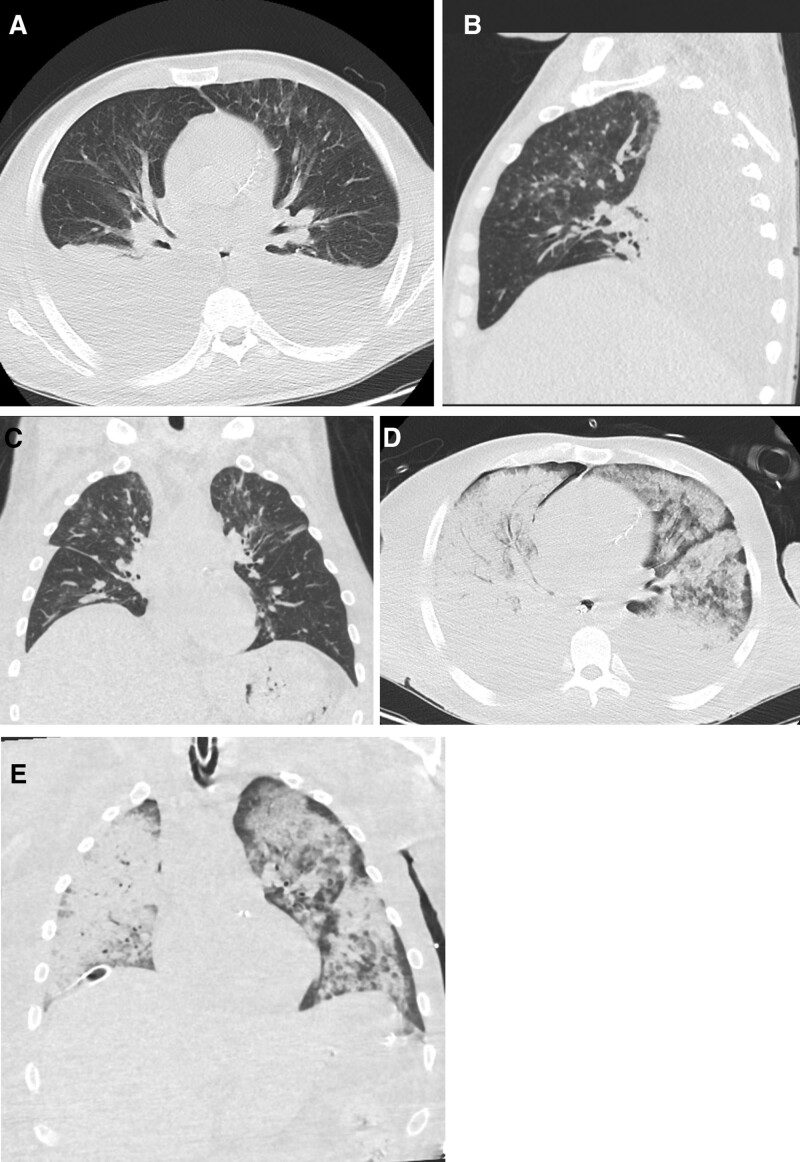
Patients confirmed positive for COVID 19, first day at admission CT chest showed. (A) Axial CT chest cuts showed moderate to severe bilateral pleural effusion with underlying consolidation collapse. (B and C) Sagittal and coronal reformate showed diffuse patchy areas of ground glassing associated with interstitial marking. (D and E) Axial and coronal images in follow up after 29 d showed development of ARDS and right sided pneumothorax with chest tube in-place. Also noted the presence of bilateral moderate to severe pleural effusion. ARDS = acute respiratory distress syndrome. CT = computed tomography.

Laino et al found that more severe disease was seen in patients with bilateral multi-lobar affection and the association of ground glassing with consolidation. This was explained by Meiler et al, who proved in a multivariable study that increased the extent of disease seen as pleural effusion, crazy paving, and geographic shape of opacifications, which are features of ARDS and can be seen in severe cases of COVID-19 pneumonia and poor prognosis (Fig. 5).

**Figure 6.**
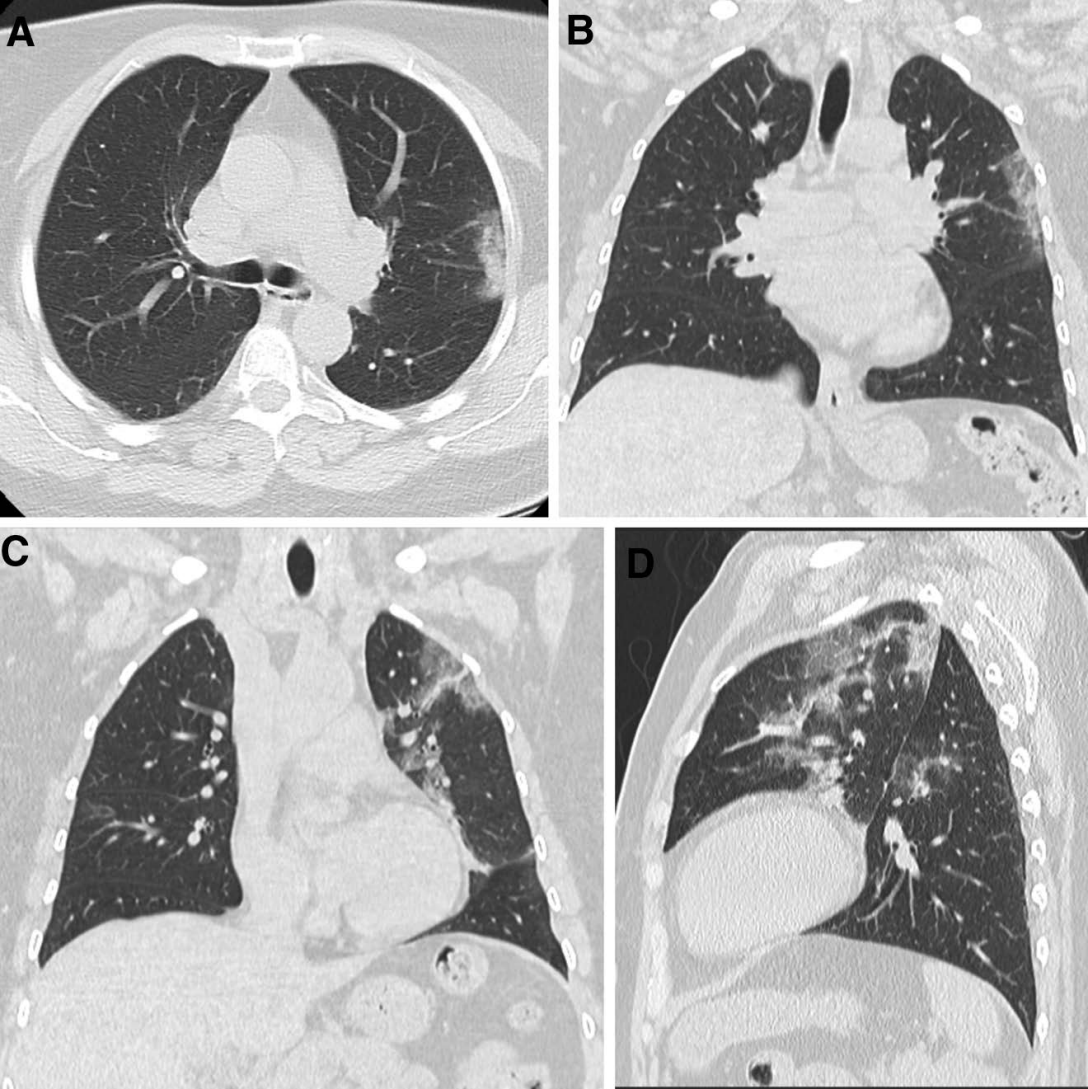
Patients confirmed positive for COVID 19, first day at admission CT chest showed. (A) Axial CT chest cuts showed left upper lung lobe sub-pleural patchy area of ground glassing. (B) Coronal reformate confirmed the left upper lung lobe mono-segmental affection. (C and D) coronal and sagittal images in follow up after 7 d showed progression of the CT chest manifestations in form of developed more patchy areas of ground glassing with development of interstitial marking. CT = computed tomography.

Only 16 patients showed progression of their chest CT after short-term follow-up, yet all had a prolonged hospital stay followed by clinical resolution and discharge (Fig. 6). In our study, the main significant factor associated with disease progression was upper lobe involvement (*P* = .044). early progression, followed by resolution and discharge, has not been discussed in previous studies. Monosegmental affection could be added to favorable outcomes even if patients show short-term CT progression, as it will be followed by clinical regression of symptoms and resolution.

**Figure 7.**
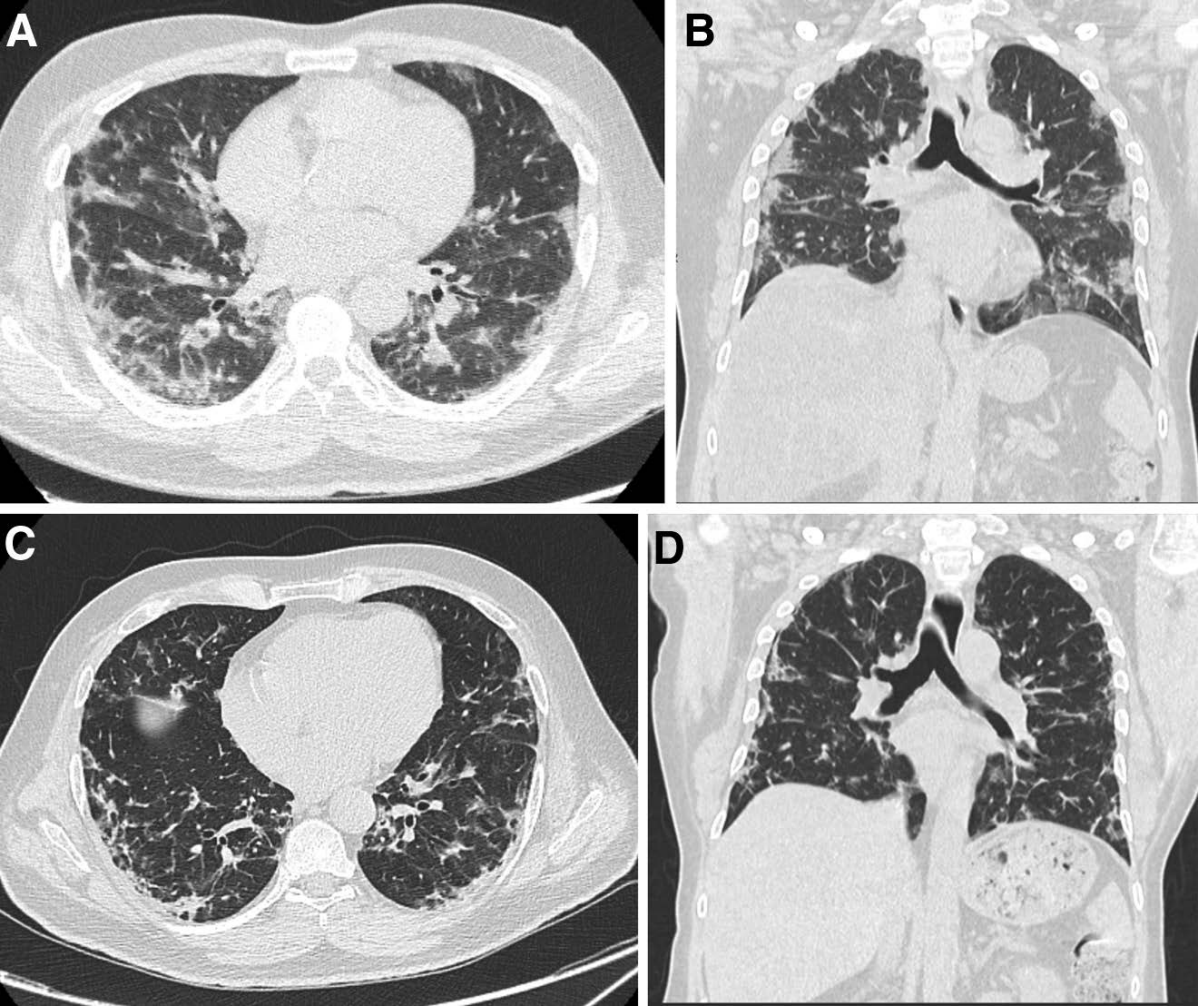
Patients confirmed positive for COVID 19, first day at admission CT chest showed. (A) Axial CT chest cuts showed bilateral multi-segmental sub-pleural patchy areas of consolidations with interstitial marking and fibrotic parenchymal bands (B) coronal reformate confirmed the bilateral multi-segmental sub-pleural patchy areas of consolidations with interstitial marking and fibrotic parenchymal bands (C and D) axial and coronal images in follow up after 16 d showed resolution of consolidation patches yet with residual interstitial marking, sub-pleural line and fibrotic parenchymal bands. CT = computed tomography.

Residual interstitial patterns were observed in 27 of 112 patients in the form of residual reticulation, ground glassing, and fibrotic-like changes. A significant factor was interstitial thickening noted at the time of admission (*P* = .017) (Fig. 7).

The main drawbacks in our study were: first, chest CT could not be repeated in patient with progressive CT course when they showed regression or resolution of clinical symptoms. Second, patients with residual fibrotic CT features should undergo intermediate- or long-term follow-up. And finally the study need to be multicenter assessment with longer follow up and larger number of patients.

In conclusion initial CT at the time of admission can help predict follow-up CT outcomes. As discussed, complete resolution was significantly associated with oligo-segmental affection, and a regressive course of CT features was observed in patients who developed CT nodular shadows. However, significant fatality was observed in patients who developed ARDS and in patients with pleural effusion.

## Author contributions

**Investigation:** Eman F. Dola.

**Methodology:** Mona Gamalludin Alkaphoury.

**Writing – original draft:** Eman F. Dola.

**Writing – review & editing:** Osama Lamie Nakhla, Mona Gamalludin Alkaphoury.
